# How contaminated is flatfish living near World Wars’ munition dumping sites with energetic compounds?

**DOI:** 10.1007/s00204-024-03834-y

**Published:** 2024-08-19

**Authors:** Edmund Maser, Tobias H. Buenning, Jennifer S. Strehse

**Affiliations:** Institute of Toxicology and Pharmacology for Natural Scientists, University Medical School Schleswig-Holstein, Brunswiker Str. 10, 24105 Kiel, Germany

**Keywords:** Submerged munitions, Energetic compounds, TNT toxicity, TNT carcinogenicity, Sediment, Flatfish

## Abstract

Seas worldwide are threatened by an emerging source of pollution as millions of tons of warfare materials were dumped after the World Wars. As their metal shells are progressively corroding, energetic compounds (EC) leak out and distribute in the marine environment. EC are taken up by aquatic organisms and pose a threat to both the marine ecosphere and the human seafood consumer because of their toxicity and potential carcinogenicity. Here, sediment samples and fish from different locations in the German North Sea of Lower Saxony were examined to determine whether EC transfer to fish living close to munition dumping areas. EC were found in sediments with a maximum concentration of 1.5 ng/kg. All analyzed fish muscle tissues/fillets and bile samples were positive for EC detection. In bile, the max. EC concentrations ranged between 0.25 and 1.25 ng/mL. Interestingly, while detected TNT metabolites in the muscle tissues were in concentrations of max. 1 ng/g (dry weight), TNT itself was found in concentrations of up to 4 ng/g (dry weight). As we found considerable higher amounts of non-metabolized TNT in the fish muscle, rather than TNT metabolites, we conclude an additional absorption route of EC into fish other than per diet. This is the first study to detect EC in the edible parts of fish caught randomly in the North Sea.

## Highlights

1. EC leak from corroding munitions and were detected in marine sediments
2. EC from corroding munition items transfer into marine biota such as fish
3. TNT mainly enters the flatfish through a route other than diet
4. EC were detected in fish fillet and posing an exposure route to human seafood consumers

## Introduction

Since the First World War, munitions have been entering the seas worldwide on a ton scale in various ways: During wartime operations, ammunition was fired for defense or attack, deployed as barrage weapons such as sea mines or for anti-submarine defense. In addition, munitions entered the sea through shipwrecks, shot down aircrafts, leftover bombs that were jettison by aircraft to ensure a safe landing at home airports and, above all, dumping by the Allies at the end of the Second World War (Lotufo et al. 2017; Böttcher et al. 2011; Rodacy et al. 2001).

While the global total amount of discarded military munitions is hardly to quantify (Beddington and Kinloch 2005), some 1.6 metric million tons of dumped conventional munitions are suspected to lie in the German coastal waters of the North and Baltic Seas (Böttcher et al. 2011). Unfortunately, sea dumping was not only performed in definite areas, but it was also common practice to discard this hazardous material on the way to the intended dumping sites (Böttcher et al. 2011). These World War relict munitions in the oceans can still explode today and therefore pose a danger for shipping and fisheries, as well as an increasing safety issue for nearshore and offshore activities, such as the construction of wind farms and the laying of cable routes and pipelines.

A new and emerging hazard is the finding that the metal shells of dumped munitions corrode on the seafloor, thereby releasing toxic energetic compounds (EC) into the marine environment. Distribution of EC and other munition-related chemicals into the marine environment has meanwhile been well documented for several dumping sites throughout the world, resulting in contamination of surface and ground waters, soils, and sediment (Talmage et al. 1999; Bełdowski et al. 2016; Edwards et al. 2016; Silva and Chock 2016; Jurczak and Fabisiak 2017; Porter et al. 2011; den Otter et al. 2023).

**Fig. 1:**
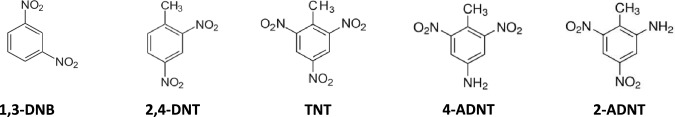
Chemical structures of 1,3-dinitrobenzene (1,3-DNB), 2,4-dinitrotoluene (2,4-DNT), 2,4,6-trinitrotoluene (TNT), 4-amino-2,6-dinitrotoluene (4-ADNT), and 2-amino-4,6-dinitrotoluene (2-ADNT)

The EC 2,4,6-trinitrotoluene (TNT) is one of the most commonly used explosives in the world and known to bioaccumulate. TNT is toxic to aquatic organisms and is shown to be absorbed by aquatic organisms (Beck et al. 2019; Maser and Strehse 2020; Strehse et al. 2017; Appel et al. 2018). It therefore poses a hazard to both the marine ecosphere and the human seafood consumer. TNT as the parent compound leaching from corroding munitions or from free-lying chunks of hexanite (German “Schiesswolle” consisting of 45–67% TNT, 5–24% hexanitrodiphenylamine and 16–25% aluminum powder) undergoes metabolic transformation processes to its main metabolites 2-ADNT (2-amino-4,6-dinitrotoluene), 4-ADNT (4-amino-2,6-dinitrotoluene), and 2,4-DNT (2,4-dinitrotoluene) (Goodfellow et al. 1983; Beck et al. 2018) (Fig. 1) by microorganisms living in the seafloor sediment or on the surface of the biota, or by metabolic activities of detoxification enzymes in the target species (Beck et al. 2018; Strehse et al. 2020). Another TNT derivative, 1,3-DNB (1,3-dinitrobenzene) often appears in parallel to TNT in environmental samples and is either a byproduct during the synthesis of TNT or an alternative energetic compound in munitions that retains high toxicities (Sunahara et al. 2009). Because TNT and its derivatives are known for their toxicity, mutagenicity and potential carcinogenicity (Sabbioni and Rumler 2007; Bolt et al. 2006; Talmage et al. 1999), chronic and persistent contamination of the marine ecosystem may cause adverse effects to all marine life, and directly affect human health via entry into the marine and human food chain (Maser and Strehse 2021; Beddington and Kinloch 2005). To date, little is known about the distribution, accumulation, and toxicokinetics of EC in marine animals, especially in seafood species (Lotufo et al. 2016; Ballentine et al. 2015; Rosen and Lotufo 2007a, 2007b; Nipper et al. 2001; Sunahara et al. 2009). Recent studies have reported the in situ occurrence of TNT and derivatives thereof in various marine animals, including blue mussels and in the bile of commercially important fish species, which raises the issue of food safety (Koske et al. 2020; Appel et al. 2018; Strehse et al. 2017; Kammann et al. 2024; Maser et al. 2023b; Porter et al. 2011).

In the present study, sediment samples and fish were examined in defined areas of the North Sea coastline of the state Lower Saxony, Germany, to determine whether EC are present in these areas and whether EC transfer to fish living in the same regions. Of special interest was to infer if EC are present in the fish muscle, which is for humans the edible part of the fish (fillet) and if there is a risk for humans who consume these fish contaminated with energetic compounds.

## Materials and methods

### Sampling

**Fig. 2:**
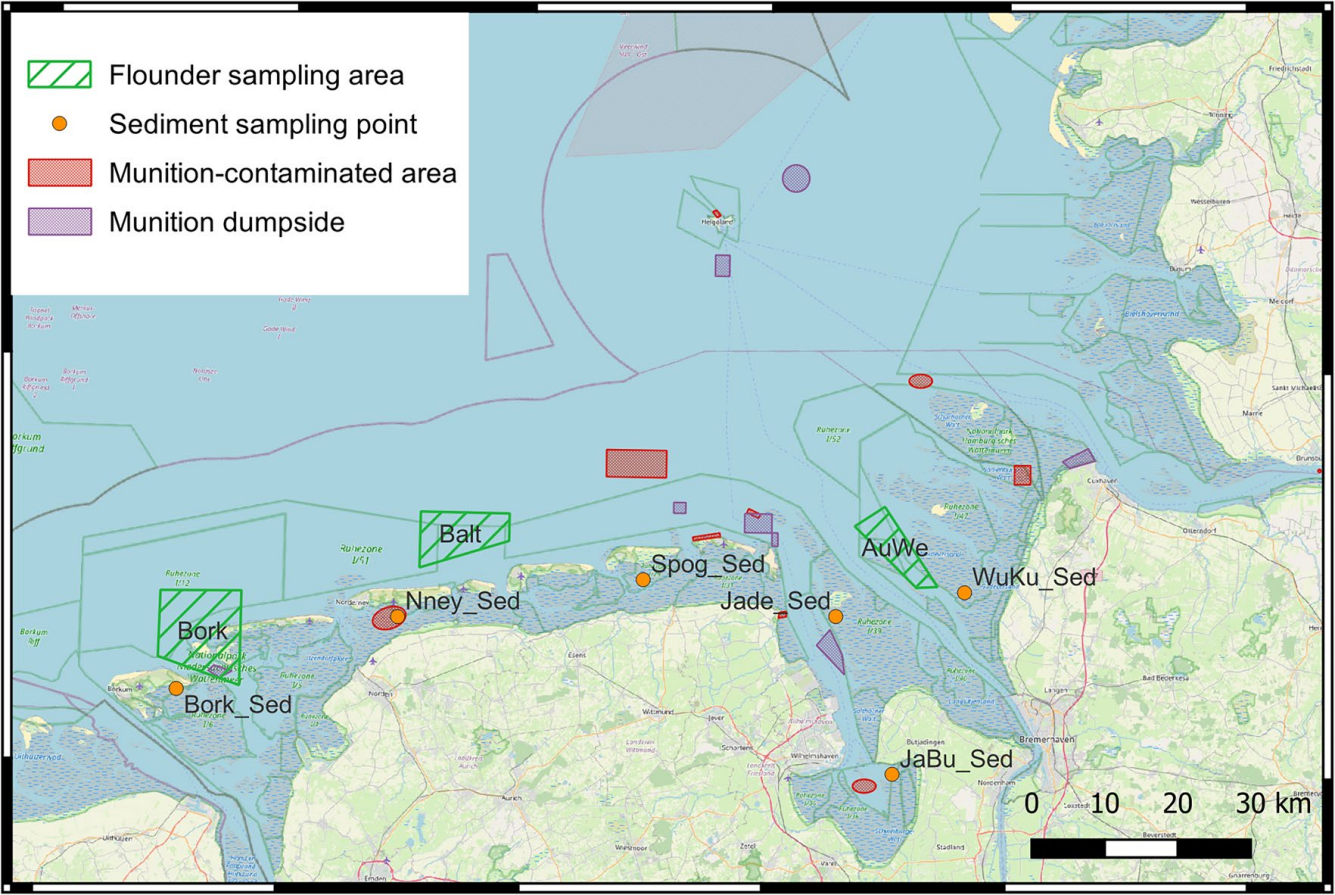
Sampling locations at the North Sea coastline of Lower Saxony/Germany for sediment (*brown spots*) and fish (*green areas*). *Bork* Borkum, *Nney* Norderney, *Spog* Spiekeroog, *Jade* Jade/Mellum, *JaBu* Jadebusen, *WuKu* Wurster Watt, *AuWe* Outer Weser, *Balt* Baltrum

The sediment samples were taken by the Lower Saxony State Office for Water Management, Coastal and Nature Conservation (NLWKN) as part of their annual routine biota monitoring. The sampling was done in August 2020 in the Wurster Watt, Jade/Mellum, Jadebusen, and Norderney regions and in September 2020 in the areas of Spiekeroog and again Jadebusen. Due to bad weather condition, sampling in the Borkum region was performed in October 2020. In 2021, sediment sampling was performed in August in the Jadebusen and Norderney region, Spiekeroog in September, and due to bad weather conditions, the region Wurster Watt in November, Jade/Mellum, and Borkum in December (Fig. 2). The sediment samples were taken from the surface by hand with a shovel to a depth of approx. 5 cm and 50 to 100 g of each sample were frozen at −20 °C as quickly as possible. Likewise, the flatfish (Flounders; *Platichthys flesus*) were caught by the NLWKN in areas close the islands of Borkum and Baltrum, and the Outer Weser in July 2019 (Fig. 2) and immediately frozen at −20 °C. The samples were delivered frozen to the Institute of Toxicology at Kiel University Medical School (Germany) for chemical analysis of energetic compounds using LC–MS/MS and GC–MS/MS.

### Chemical analyses

#### Materials and chemicals

For calibration, 2,4,6-trinitrotoluene (98.9% purity, 1 mg/mL, in acetonitrile (ACN):methanol (MeOH) 50:50), 1,3-dinitrobenzene (97.0% purity, 1 mg/mL, in ACN:MeOH 50:50), 2,4-dinitrotoluene (98.3% purity, 1 mg/mL in ACN:MeOH 50:50), 4-amino-2,6-dinitrotoluene (98.4% purity, 1 mg/mL, in ACN:MeOH 50:50), and 2-amino-4,6-dinitrotoluene (97.8% purity, 1 mg/mL, in ACN:MeOH 50:50) were purchased from AccuStandard, New Haven, USA. For spiking, isotopically labeled TNT (^13^C7, 99%; ^15^N3, 98%, 1 mg/mL in benzene, wetted with > 33% H_2_O) was purchased from Cambridge Isotope Laboratories, Inc, Andover, USA. Acetonitrile (UHPLC-grade, purity ≥ 99.97%) was purchased from Th. Geyer (Renningen, Germany) and used without further purification. CHROMABOND Easy polystyrene-divinylbenzene-copolymer reversed-phase solid-phase extraction columns 80 µm, 3 mL/200 mg and 1 mL/30 mg (Macherey Nagel, Düren, Germany) were used. Ultrapure water (18.2 MΩ cm) was prepared on site with a Veolia ELGA Purelab Flex system (Veolia Water Technologies Deutschland GmbH, Celle, Germany). *β-*Glucuronidase type H-1 from *Helix pomatia* was used (Sigma-Aldrich Chemie GmbH, Taufkirchen, Germany).

#### Sediment preparation

Sediment samples were extracted as described in Bünning et al. (2021). In brief, 100 g wet sediment was mixed with 250 mL ultrapure water, spiked with 25 ng ^13^C^15^N-TNT, shaken for 80 min and sonicated for 15 min. Samples were then centrifuged at 4500 rpm (10 °C) for 15 min (J2-HS centrifuge, Beckman Coulter GmbH, Krefeld, Germany), filtered through a 595 1/2 pleated filter and applied onto SPE columns (Macherey–Nagel™ Chromabond™ Easy, Düren, Germany) using a mild vacuum. Columns were dried for 30 min i.vac., eluted with 4 mL ACN, concentrated to 1000 µL and stored for GC–MS/MS analyses at −20 °C in 1.5 mL amber vials. For LC–MS analysis, 250 µL of samples in ACN were diluted with 750 µL water (LC–MS quality, spiked with 1 mM ammonium acetate) and filtered through 0.2 µm syringe filters into 1.5 mL amber vials and stored at −20 °C until further use.

#### Flatfish preparation (Flounders; *Platichthys* flesus)

A total of 33 muscle and bile samples were examined for EC. Of these, nine samples were from the Borkum fishing area, four from the area near the island of Baltrum, and 20 from the Outer Weser fishing region. To collect the tissue samples, the flounders were thawed, measured, and weighed; after then, the EC were extracted from the muscle and bile samples for analysis using GC–MS/MS.

The bile was collected from gall bladder with disposable needles (0.15 mm × 35 mm) and syringes (1 mL), and transferred into cryovials before snap freezing in liquid nitrogen. For EC analyses, the method of Ek et al. (2006) was adapted to process the bile samples. In brief, 25 µL of bile was added to a 1.5 mL microreaction tube containing 900 units of *β*-glucuronidase from *Helix pomatia* dissolved in 100 µL sodium acetate buffer (pH 4.8). An amount of 2.5 µL of an isotope standard were added (^13^C-1,3DNB, ^13^C,^15^N-TNT, 4-ADNT-d5, 2-ADNT-d5; 100 ng/mL in ACN). Samples were incubated at 37 °C for 20 h in a Thermomixer Compact (Eppendorf, Hamburg, Germany). After cooling to room temperature, the samples were loaded onto 1 mL Chromabond Easy columns (preconditioned with 300 µL ultrapure water, 600 µL MeOH, and 300 µL ultrapure H_2_O). The microreaction tubes were rinsed three more times with 500 µL of ultrapure water each, which was also given onto the columns. Columns were dried i. vac. for 15 min, followed by elution with five times 50 µL ACN. The eluate was transferred into 1.5 mL amber glass vials with 250 µL glass inserts and stored at −80 °C.

One fillet per fish was dissected and stored at −20 °C. Fish muscle samples were processed as described for blue mussel samples in Bünning et al. (2021). In brief, muscle samples were freeze-dried for 48–72 h (dry weight = 22 ± 4% of wet weight). Lyophilized samples were homogenized using mortar and pestle, and aliquots of 1g were weighed into 15 mL tubes. Five mL of ACN and 10 µL of a 100 ng/mL isotope standard (^13^C-1,3DNB, ^13^C,^15^N-TNT, 4-ADNT-d5, 2-ADNT-d5; in ACN) was added as internal standard. Samples were mixed for 60 s, sonicated for 15 min, and centrifuged at 4100 rpm (4 °C) for 10 min. Supernatants were transferred into 50 mL graded flasks, diluted with ultrapure water, and applied onto unconditioned Chromabond Easy SPE-columns using mild vacuum. Columns were then dried i.vac for 30 min, and samples were eluted with three times 1 mL ACN, concentrated to 600 µL, and stored at −80 °C in 1.5 mL amber vials.

#### GC–MS/MS analysis

A Thermo Scientific TRACE 1310 gas chromatograph coupled to a TSQ 8000 EVO triple quadrupole mass spectrometer with electron ionization source was used in selective reaction monitoring (SRM) mode. The GC was equipped with a TraceGold TG-5MS amine 15 m × 0.25 mm × 0.25 µm column (Thermo Fisher Scientific Inc, Waltham, MA, USA). For sediment samples, splitless injections on a split-/splitless-injector were performed on quartz wool injection port liners (4 mm × 6.5 mm × 78.5 mm, Thermo Fisher Scientific Inc, Waltham, MA, USA). Injections of biota samples were carried out on a programmable temperature vaporization (PTV)-injector with packed quartz wool liners (2 mm × 2.75 mm × 120 mm, Thermo Fisher Scientific Inc, Waltham, MA, USA). Helium served as carrier gas for the GC, and Argon as collision gas for the mass spectrometer (both Alphagaz, purity 99.999%). After injection of 5 µL onto quartz wool liner at 70 °C, the solvent was removed in the carrier gas stream. The analytes were then applied to the column by increasing the injector temperature (5 °C s^−1^ to 240 °C). The initial oven temperature of 100 °C was maintained for 1.0 min, then increased to 220 °C at 35 °C min^−1^, and baked out at 70 °C min^−1^ at 280 °C after 0.7 min until the end of the total measurement time (6.99 min). Spectra were recorded and analyzed in Chromeleon 7.2 (Thermo Fisher Scientific Inc, Waltham, MA, USA). Retention times and (SRM) transitions were determined using purchased standards and are shown in Table 1.

**Table 1:** GC–MS/MS retention times and quantitative (Q) and qualitative (q) SRM transitions of the compounds examined

Compound	Retention time (min)	Precursor ion (m/z)	Quantitation ion (m/z)	Collision energy (eV)	Type
1,3-DNB	2.90	122.0	75.0	12	Q
		168.0	75.0	20	q
		168.0	122.0	8	q
2,4-DNT	3.26	165.0	63.1	22	Q
		165.0	90.1	16	q
		165.0	118.1	8	q
TNT	3.96	210.0	164.1	6	Q
		164.0	90.1	10	q
		180.1	76.1	12	q
4-ADNT	4.88	197.0	180.1	6	Q
		180.0	163.1	8	q
		163.0	78.0	14	q
2-ADNT	5.05	197.0	180.1	6	Q
		180.0	133.0	6	q
		180.0	67.0	12	q

#### LC–MS/MS analyses

A Sciex QTrap5500 triple quadrupole mass spectrometer (AB Sciex LLC, Framingham, MA, United States of America) with a Turbo V electron spray ionization ion source coupled to a UHPLC consisting of a Shimadzu Nexera LC-40D XS quaternary pump with degasser (Shimadzu Corporation, Kyoto, Japan), an Agilent 1200 G1316A column oven, and a CTC HTS PAL autosampler with a cool stack and a VICI Cheminert 6-Port injection valve, equipped with a 5 µL sample loop was used. Separation was carried out on a RESTEK Raptor Biphenyl 1.8 µm column (150 mm × 2.1 mm) with pre-column (Restek Corporation, Centre County, PA, United States of America). Spectra were recorded in Sciex Analyst 1.7.2 and analyzed in Sciex MultiQuant 3.0.3. The MS parameters were set as follows: CUR 20 psi, TEM 350 °C, GS1 40 psi, GS2 40 psi, negative polarity. The measurement started with a 5 min isocratic phase with a ratio of 40% H_2_O (containing 2.5 mM ammonium acetate) and 60% MeOH at 0.25 mL/min and 35 °C, then increased to 95% MeOH by minute 6, and maintained this ratio for 6 min. After the end of the measurement, the initial ratio was reestablished for 7 min. Measurements were carried out using selective reaction monitoring. The transitions and retention times were determined using purchased standards and are listed in Table 2.

**Table 2:** LC–MS/MS retention times and quantitative (Q) and qualitative (q) SRM transitions of the compounds examined

Compound	Retention time (min)	Precursor ion (m/z)	Quantitation ion (m/z)	Collision energy (eV)	Type
TNT	9.04	226.0 [M–H]^−^	46.0	−50	Q
			196.0	−18	q
4-ADNT	4.36	196.0 [M–H]^−^	149.0	−19	Q
			46.0	−56	q
2-ADNT	4.59	196.0 [M–H]^−^	46.0	−56	Q
			136.0	−22	q

## Results

### Chemical analyses

Method-specific detection limits (LODs, Table 3) were determined as described in Bünning et al. (2021) using solvent standards according to the EUR 28099 EN calibration standard method. The limit of quantification (LOQ) was set at 3.3 times the detection limit. The matrix specific limits of detection (LoD) and limits of quantification (LoQ) were determined using spiked matrix samples and are given in Table 4.

**Table 3:** Method-specific limits of detection of the GC–MS/MS and LC–MS/MS methods

Compound	GC–MS/MS			LC–MS/MS		
	LoD (fg/µL)	LoQ (fg/µL)	*R*^2^	LoD (fg/µL)	LoQ (fg/µL)	*R*^2^
1,3-DNB	32	105	0.9644	–	–	–
2,4-DNT	10	33	0.9934	–	–	–
TNT	47	155	0.9878	131	430	0.9730
4-ADNT	8	26	0.9959	124	412	0.9764
2-ADNT	11	37	0.9919	83	274	0.9893

**Table 4:** Matrix specific limits of detection of the GC–MS/MS and LC–MS/MS methods

EC	Muscle (GC–MS/MS)		Bile (GC–MS/MS)		Sediment (LC–MS/MS)	
	LoD (ng/g d.w)	LoQ (ng/g d.w)	LoD (ng/mL)	LoQ (ng/mL)	LoD (ng/kg)	LoQ (ng/kg)
1,3-DNB	0.02	0.06	0.08	0.26	0.8	2.6
2,4-DNT	0.02	0.06	0.03	0.08	0.2	0.5
TNT	0.10	0.39	0.12	0.39	0.33	1.1
4-ADNT	0.03	0.09	0.02	0.07	0.31	1.0
2-ADNT	0.02	0.07	0.03	0.09	0.21	0.68

### Distribution of EC in sediment samples

EC could actually be detected in some of the collected sediments from Lower Saxony (Fig. 2) (Table 5). The measured concentrations were mostly in the trace range below the limit of detection (LoD), or below the limit of quantification (LoQ) but above the LoD (Tables 4 and 5). For example, in sediment, a concentration for 1,3-dinitrobenzene (1,3-DNB) of less than 0.8 ng/kg, for TNT a concentration of higher than 0.33 but less than 1.1 ng/kg, for 4-amino-2,6-dinitrotoluene (4-ADNT) a concentration of higher than 0.31 but less than 1.0 ng/kg, and for 2-amino-4,6-dinitrotoluene (2-ADNT) a concentration of higher than 0.21 but less than 0.68 ng/kg was detected (Table 4). In the samples from the Norderney, Jadebusen, and Jade/Mellum sampling regions, TNT was found in concentrations between 1.2 and 1.5 ng/kg of dried sediment. In the Norderney and Jadebusen region, 4-ADNT was also detected in concentrations of 1.5 and 1.3 ng/kg of dried sediment, respectively. The samples from the Wurster Watt and Spiekeroog region showed no evidence of EC.

**Table 5:** EC abundance in sediment samples from Lower Saxony

	1,3-DNB	2,4-DNT	TNT	4-ADNT	2-ADNT
Norderney	<LoD	<LoD	1.5 ng/kg	1.5 ng/kg	> LoD < LoQ
Borkum	<LoD	<LoD	<LoD	<LoD	> LoD < LoQ
Wurster Watt	<LoD	<LoD	<LoD	<LoD	<LoD
Spiekeroog	<LoD	<LoD	<LoD	<LoD	<LoD
Jadebusen	<LoD	<LoD	1.2 ng/kg	1.3 ng/kg	> LoD < LoQ
Jade/Mellum	<LoD	<LoD	1.3 ng/kg	<LoD	<LoD

Analysis of sediment samples was performed by GC–MS/MS technology. Abbreviations: 1,3-DNB (1,3-dinitrobenzene), 2,4-DNT (2,4-dinitrotoluene), TNT (2,4,6-trinitrotoluene), 4-ADNT (4-amino-2,6-dinitrotoluene), 2-ADNT (2-amino-4,6-dinitrotoluene), (LoD) limit of detection, (LoQ) and limits of quantification

### EC concentrations in fish bile

**Fig. 3:**
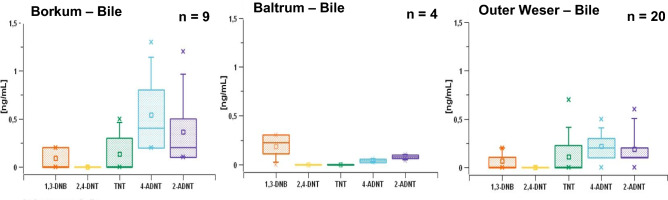
EC concentrations in flatfish bile from Lower Saxony (North Sea, Germany). Analysis was performed by GC–MS/MS technology. *1,3-DNB* 1,3-dinitrobenzene, *2,4-DNT* 2,4-dinitrotoluene, *TNT* 2,4,6-trinitrotoluene, *4-ADNT* 4-amino-2,6-dinitrotoluene, *2-ADNT* 2-amino-4,6-dinitrotoluene. Statistics and figures were created with SciDAVis 2.7

A total of 33 bile samples were examined for different EC. Of these, nine samples were from the Borkum area, four from the area near the island of Baltrum and 20 from the Outer Weser sampling region (Fig. 2). All flatfish bile samples were positive for EC detection (Fig. 3): maximum concentrations found were 0.25 ng/mL 1,3-DNB, 0.50 ng/mL TNT, 1.25 ng/mL 4-ADNT, and 1.2 ng/mL 2-ADNT in the flatfish bile of the Borkum region, while bile samples from the Baltrum region contained 2- and 4-ADNT well below 0.25 ng/mL each and 1,3-DNB of approx. 0.25 ng/mL. The flatfish caught in the Outer Weser contained up to 0.25 ng/mL of 1,3-DNB, 0.75 ng/mL of TNT, and 0.5 ng/mL or 0.6 ng/mL of 4- and 2 -ADNT, respectively, in bile samples.

### EC concentrations in flatfish muscle samples

**Fig. 4:**
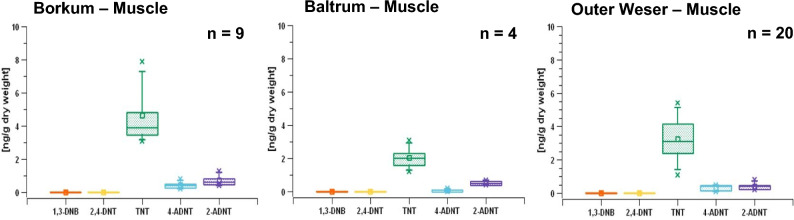
EC concentrations in flatfish muscle samples from Lower Saxony (North Sea, Germany). Analysis was performed by GC–MS/MS technology. *1,3-DNB* 1,3-dinitrobenzene, *2,4-DNT* 2,4-dinitrotoluene, *TNT* 2,4,6-trinitrotoluene, *4-ADNT* 4-amino-2,6-dinitrotoluene, and *2-ADNT* 2-amino-4,6-dinitrotoluene. Statistics and figures were created with SciDAVis 2.7

A total of 33 muscle samples were examined for different EC. Of these, nine samples were from the Borkum area, four from the area near the island of Baltrum and 20 from the Outer Weser sampling region (Fig. 2). EC residues could be measured in the muscle samples of the flatfish. The TNT metabolites 2- and 4-ADNT were measured in all three geographical regions in concentrations of a maximum of 1 ng/g of dry weight (Fig. 4). TNT itself was found in the samples (mean per dry weight) around 4 ng/g in the Borkum region, 2 ng/g in the Baltrum region and 3 ng/g in the Outer Weser region (Fig. 4).

## Discussion

In the present study, muscle and bile samples from a total of 33 flatfish were examined for different EC. The study is an expansion of the investigation reported in Maser et al. (2023b) to include a larger geographical area and dump sites, and the results from the present study are consistent with those in Maser et al. (2023b). The mean concentrations measured in bile for each of the EC (TNT, 2-ADNT, 4-ADNT, 2,4-DNT, and 1,3-DNB) were between 0 and 1.25 ng/mL, with higher concentrations of 4- and 2-ADNT than of TNT itself. Koske et al. (2020) also detected EC in the one- to two-digit ng/mL range in the bile of flatfish from the Kolberger Heide, which is a munition dumping area in the western Baltic Sea in Germany. Comparable to our results, the TNT metabolites 4-ADNT (mean 17.06 ng/mL) and 2-ADNT (mean 1.60 ng/mL) were found in higher concentrations, while TNT concentrations were low (around 0.1 ng/mL) (Koske et al. 2020).

In our investigation, EC residues could also be measured in the muscle samples of the flatfish. While the TNT metabolites 2- and 4-ADNT were measured in concentrations of a maximum of 1 ng/g (dry weight), TNT itself was found in the flatfish muscle (mean per dry weight) of 4 ng/g in the Borkum region, 2 ng/g in the Baltrum region, and 3 ng/g in the Außenweser region. Hence, TNT accumulated at higher concentrations in muscle tissue than in the bile. This is interesting, because we have here a reversal of the ratios of unmetabolized TNT compared to its metabolites 2- and 4-ADNT in muscle versus bile. From this, it could be deduced that TNT can accumulate better in the muscle than in the bile, and that there is an alternative uptake of EC into the muscle apart from the uptake via diet. Lotufo (2011) exposed Sheepshead minnows (*Cyprinodon variegatus*) to radiolabeled isotopes of TNT and detected in total 46% of the TNT metabolites ADNTs resided in the liver and 64% of the parent compound in the viscera. Tissue-specific concentrations were determined with 6 µmol/kg for liver versus 280 µmol/kg in the viscera (Lotufo 2011). Since bile is produced in the liver, similar ratios of TNT to ADNTs should be expected in both compartments.

Further evidence that fish accumulated TNT via the dietary route were provided by Belden et al. (2005), who exposed channel catfish (*Ictalurus punctatus*) via food pellets containing different concentrations of TNT, and Lotufo and Blackburn (2010), using the amphipods *Leptocheirus plumulosus* as prey and the fish *Cyprinodon variegatus* as predator. Houston and Lotufo (2005) exposed the oligochaete worm *Lumbriculus variegatus* to ^14^C-labeled TNT for 5 h in water and, after frozen into meal-size packages, fed them to individual juvenile fathead minnows (*Pimephales promelas*).

As a matter of fact, xenobiotics that are ingested into organisms via diet are absorbed from the intestinal tract into the venous blood stream and distribute in various tissues. Depending on their lipophilicity, these compounds may accumulate in marine organisms along the marine food chain. According to the principles of toxicokinetics, these substances undergo a first pass metabolism in the liver or intestine which leads to the excretion of the metabolites via the bile or urine, or their redistribution within different organs and/or tissues via the blood stream. For example, the TNT metabolites ADNTs and DANT were found in various fish organs, especially DANT in the liver (Beck et al. 2022). Mariussen et al. (2018) showed that TNT is excreted by salmon through the gall bladder, and that TNT transformation products accumulate in bile. This is the reason why bile samples of the fish investigated show higher concentrations of the TNT metabolites 2-ADNT and 4-ADNT compared to TNT (Lotufo 2011) and corresponds to the findings of Ek et al. (2008) who detected mainly 2- and 4-ADNT in fish bile rather than TNT itself. Ownby et al. (2005) found that TNT metabolite accumulation in fish viscera during aqueous exposure was higher than TNT (Ownby et al. 2005). From our results, that fillet contains higher concentrations of non-metabolized TNT compared to both ADNT metabolites, lead us to conclude that TNT enters the fish through additional routes other than diet, possibly through gills. Organ-specific uptake and depuration in Atlantic salmon (*Salmo salar*) exposed to TNT was studied by Mariussen et al. (2018). They indeed found that TNT is taken up primarily by the gills and rapidly excreted from fish via the bile. Importantly, TNT and the metabolites 2-ADNT and 4-ADNT were found in the muscle tissue, whereas only 2-ADNT and 4-ADNT were found in the bile.

As fish gills represent the major interface between water and the body of a fish, and are strongly perfused with blood, a xenobiotic transfer may occur across the gill lamellae, such that the branchial route should be considered as a toxicant uptake. It has been demonstrated that there is a significant relationship between toxicant uptake and fish oxygen uptake regardless of chemical hydrophobicity and fish species. These results support the view that the main route of toxicant entry for fish is across the gills, where gas exchange occurs. Exchange across the gills is fast and toxicant intake via other sources, e.g., feeding, is much less important (Yang et al. 2000) than generally postulated for water breathing animals. This fits with previous own research that also found higher TNT concentrations in the fillets compared to bile samples in fish caught near a munitions-containing shipwreck (Maser et al. 2023b).

Aquarium studies showing TNT uptake in fish via contaminated water were performed by Lotufo et al. (2016), Yoo et al (2006), and Lotufo and Lydy (2005), while the present study was a field study taking sediment contamination into account. Flounders (*Platichthys flesus*) are a group of flatfish species that are found at the ocean floor and hide themselves into the sediment as protection against predators. Sediments of dumping sites that contain EC may therefore serve as source of EC contamination of flatfish. Studies investigating sediment as route of exposure (Lotufo et al. 2010) concluded that direct contact with the sediment bed or resuspended sediment is a not relevant route of EC exposure to near-bottom fish. Other studies provided no evidence that sediment contamination with EC is a causative factor for the induction of adverse biological effects in near-bottom fish (Bernet et al. 2011; Rosen and Lotufo 2010). While the body burden of the fish of Lower Saxony with TNT and its metabolites were obvious (Figs. 3 and 4), there were no signs of biological malfunctions visible. In the present investigation, the measured concentrations in sediment samples were near the LoQ (Table 4) at a maximum level of 1.5 ng/kg sediment (Table 5), thereby resembling EC concentrations near the John Mahn wreck site in Belgian waters (Maser et al. 2023b). Higher concentrations (in the ng per g range) have been measured in the Kolberger Heide in the Baltic Sea of Germany (Jansen et al. 2011) or in Eastern Scheldt in The Netherlands (den Otter et al. 2023).

Laboratory studies reveal that EC, especially TNT and its metabolites, have acute and chronic negative effects on various marine species. With regard to fish, a variety of acutely toxic concentrations have been described. Koske et al. (2019) determined an LC50 of 4.5 mg/L for TNT in zebrafish embryos, as well as 13.4 mg/L for 2-ADNT and 14.4 mg/L for 4-ADNT. For the eyespot lyrefish (*Synchiropus ocellatus*), Nipper et al. (2001) described an LOEC of 10.8 mg/L TNT, based on the survival of the fish larvae. Juhasz and Naidu (2007) published an EC50 of 8.2 mg/L for TNT for the red drummer (*Sciaenops ocellatus*) and an EC50 of 2.3 mg/L TNT for the gemfish (*Cyprinodon variegatus*) based on fish mortality. Talmage et al. (1999) reported LC50 concentrations in the range of 0.8 to 3.7 mg/L TNT for four fish species in their review. The authors independently agree in their publications that fish are among the most sensitive organisms to exposure to EC. Furthermore, Lotufo et al. (2010) showed in a laboratory study that more than 90% of juvenile sheepshead minnows (*Cyprinodon variegatus*) survive a 4-day exposure to sediment spiked with 7 mg/kg TNT, but when the TNT concentration in the sediment reached 340 mg/kg did all the fish die within 24 h.

Other studies reported lethal and sublethal effects at TNT concentrations below 1 mg/L. For example, Koske et al. (2019) showed that TNT and its metabolites 2- and 4-ADNT damage the DNA of zebrafish embryos even at the lowest tested concentrations (0.1 mg/L for TNT and 1 mg/L for 2- and 4-ADNT), while Liu et al. (1983) determined lethal concentrations of TNT to fish in the range of 0.8–5.0 mg/L water. Behavioral responses of the fathead minnow, such as lethargy and loss of motor control, have also been observed after exposure to TNT for 96 h at a concentration of 0.46 mg/L (Smock et al. 1976).

For an ecotoxicological risk assessment, the actual EC concentrations in the marine environment must be taken into account. In the present study, the measured concentrations in sediment samples from Lower Saxony were a maximum of 1.5 ng/kg sediment. These concentrations are several orders of magnitude lower than the acute effect concentrations shown above for fish in laboratory studies. Seen from this perspective, the measured EC concentrations in our study do not appear to pose an acute health problem for the fish living there.

However, a direct extrapolation from laboratory studies at relatively high EC concentrations should only be done with caution, as so far little is known about the long-term effects of low EC concentrations. In this context, the so-called cocktail effect must also been considered. This phenomenon describes the enhancement of toxic properties of various individual substances or groups of substances through additive effects within an organism, even if the measured concentration of an individual substance is below its previously known effect threshold. Negative effects on fish cannot therefore be completely ruled out. Mariussen et al. (2018) examined the effects of TNT on juvenile Atlantic salmon (*Salmo salar*). Fish were exposed to dissolved TNT with an initial concentration of 1 mg/L for 48 h. At the end of the exposure experiment, the mortality of the fish was increased compared to the control. All salmon, including those that survived the experiment, were found to have severe bleeding in the back-muscle tissue near the spine, as well as effects on blood parameters, such as glucose, urea, hematocrit, and hemoglobin. The authors concluded that if the exposure period had been extended, all fish would have died from the severe effects of TNT. Leffler et al. (2014) exposed alevins of Atlantic salmon to TNT wastewater for 40 days. In the high exposure group (2.1 mg/L TNT), they observed approximately 25% mortality after 14 days and 100% mortality after 40 days. In the group exposed to 0.41 mg/L, they observed approximately 30% mortality after 40 days.

In a field study in the Kolberger Heide munitions dumping area (Kiel Bight, western Baltic Sea, Germany), low TNT concentrations between 0.5 and 51.5 ng/L were found in the water with a median concentration of 3 ng/L (Esposito et al. 2023). Interestingly, poorer health status was demonstrated in flatfish from the same area than in reference areas, with the fish also having, for example, higher rates of liver nodules and tumors (Straumer and Lang 2019).

Figure 2 shows an overview of the sampling locations in Lower Saxony with the munition-contaminated areas, suspected areas, and munition dumping areas. Several hot spots regarding the release of EC are emerging here. These include the regions in the Jade area and the Jade Bay as well as the East Frisian Islands, especially Borkum and Baltrum. In the vicinity of the Borkum and Baltrum fishing areas, estimated 2000 metric tons of mines, grenades, bombs, torpedoes, rocket-propelled grenades, small ammunition, as well as plate and sea mines were dumped in these areas (Böttcher et al. 2011). In addition, because of so-called on-route dumping at that time munition-contaminated sites can also be expected outside the declared areas. The Jade Bay and Jade collection region also overlap with a munitions-contaminated or munitions dumping area (Böttcher et al. 2011). According to estimates, between 650,000 and 1.2 million metric tons of conventional munitions are located in these areas (Böttcher et al. 2011). Only in the Wurster Watt and Spiekeroog, sampling spots are no known munition dumpsites or suspected areas within a radius of three kilometers (Böttcher et al. 2011). These were also the only areas investigated in which no evidence of EC could be found in the sediment samples in this study.

With regard to food safety, it is of interest in how far the carcinogenic EC accumulate in the edible part of sea-food species. Bioaccumulation of chemicals from one compartment to another, or from one species to another, is defined as concentrations increasing by a factor of higher than 1000-fold, while values below 1000-fold are regarded as bioconcentration (Lotufo et al. 2013; Ownby et al. 2005). Whether or not a substance has the potential to bioaccumulate is dependent on its logKow (Arnot and Gobas 2006). The bioconcentration factor for TNT varies widely from 0.3 to 9.7 mL g^−1^ for various marine and freshwater invertebrates and fish species and was until today regarded as a compound that bioconcentrates rather than bioaccumulates (Lotufo et al. 2013). However, when considering the transfer of TNT from sediment samples in the range of 1 ng/kg of sediment, or even below, on the one side, and the occurrence of TNT in the fish fillets in the range between 2000 and 4000 ng/kg, then the demand of bioaccumulation has been fulfilled in our findings. Here, it is interesting to speculate that flatfish living in and feeding from the sediment are continuously exposed to EC. Moreover, flatfish hide in the sediment and stir up sediment in search of food, thereby mobilizing EC.

The question now arises as to whether the consumption of this TNT-contaminated fish poses a health risk for humans. Particularly noteworthy here is the chronic toxic risk of consuming low-contaminated fish and seafood in terms of the potential carcinogenicity of TNT (Bolt et al. 2006). Calculations show that, even with a lifelong daily consumption of an average consumption of approx. 39 g (FIZ 2017) of the fillet of the fish examined in this project, no negative health effects should be expected for the human consumer (Maser and Strehse 2021), as the TNT concentrations were in the single-digit nanogram range per gram of dry weight.

However, this could worsen in the coming years as the corrosion of submerged munitions continues, thereby increasing the release of EC into the marine environment. Studies in the Kolberger Heide munition dumping area in the Baltic Sea near Kiel (Germany) have shown an increased uptake of EC in blue mussels exposed to free-lying chunks of hexanite (German Schiesswolle), when compared to mussels mounted near corroding moored mines with more or less intact metal shells (Appel et al. 2018; Strehse et al. 2017). From the EC concentrations found in the highly contaminated blue mussels, it was concluded that they were no longer suitable for consumption due to the carcinogenic risk (Maser and Strehse 2021). Targeted blast-in-place detonations of munitions underwater also lead to a drastic increase in EC concentrations in the surrounding sediment and water (Maser et al. 2023a), which may also enhance the contamination of fish living in the nearby area.

In summary, the present investigations in the areas of the Lower Saxony North Sea showed that there is a correlation between munition deposits and the occurrence of EC in sediment and flatfish living there and that a transfer of EC from the munition items into the fish is obvious. So far, the EC concentrations found in the sediments and flatfish are low, but due to live-long exposure, there is a risk that the fish themselves will experience negative effects on their health. Whatsoever, the EC concentrations in the fillet as the edible portion of the fish are so low that there is no danger to humans if they consume these fish. However, the further corrosion of the munition bodies could lead to toxic levels of EC in fish in the future. This would particularly affect flatfish, which are relatively stationary and prefer to stay in the sediments of the seabed.

## Data Availability

Data are contained within the article.
